# Clinical and Histopathologic Investigation of Periapical Actinomycosis with Cutaneous Lesion: a Case Report

**Published:** 2015-09

**Authors:** Davood Jamshidi, Fariborz Moazami, Fereshteh Sobhnamayan, Ali Taheri

**Affiliations:** a Dept. of Endodontics, School of Dentistry, Qazvin University of Medical Sciences, Qazvin, Iran.; b Dept. of Endodontics, School of Dentistry, Shiraz University of Medical Sciences, Shiraz, Iran.; c Dept. of Pathology, School of Dentistry, Kerman University of Medical Sciences, Kerman, Iran.

**Keywords:** Actinomycosis, Apicoectomy, Cutaneous Sinus Tract, Misdiagnosis

## Abstract

Management of an extra-radicular infection is a challenging procedure that requires surgical intervention. This report describes a patient with discharging cutaneous lesion that required apical surgery. A 40-year-old woman was referred to the Department of Endodontics, Shiraz Dental School with chief complaint of a cutaneous sinus tract. She had been treated by a dermatologist and an otolaryngologist. The patient had also received orthograde root canal treatment of tooth #16. Yet, the lesion was still discharging and the patient was scheduled for surgery. Histopathologic analysis of the lesion showed actinomycosis infection. A 36-month follow-up revealed clinical and radiographic healing.

## Introduction

Apical periodontitis is an inflammatory disease which is formed in response to intraradicular infection(1) and usually constitutes a barrier against the spread of infection to the bone.(2)

Microorganisms are not usually present in apical periodontitis lesions,(3) but in an extra-radicular infection, bacteria can invade the inflamed periradicular tissues.(4) In such conditions, extra-radicular bacteria have been found in biofilms adhering to the apical root surface(5) or located within the body of inflammatory lesion, usually forming cohesive actinomycotic colonies.(6)

The extra-radicular infection can be dependent on or independent of the intraradicular infection. For example, acute apical abscess is mostly dependent on intraradicular infection(4)and periapical actinomycosis is an independent extra-radicular infection(2) caused by anaerobic or facultative gram-positive, non-spore-forming, filamentous rod bacteria. These bacteria are not virulent and produce chronic, slow-developing, opportunistic granulomatous infections.(7) Strains of actinomyces have been associated with cases of failed endodontic therapy.(8)

This paper reports a case of extra-radicular actinomycosis that caused failure of endodontic treatment, and thus made apical surgery necessary. 

## Case Report

A 40-year-old Afghan female patient was referred to the Endodontic Department of Shiraz Dental School by an endodontist. She was complaining about a persistent cutaneous lesion on the right cheek of her face persistent for more than a year.

**Figure 1:**
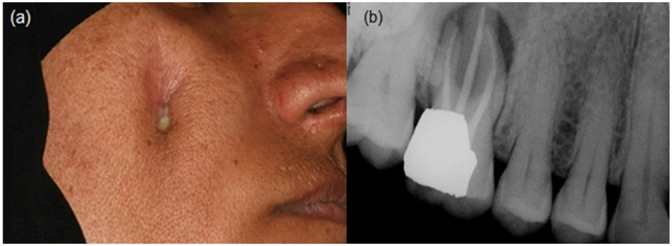
Radiograph and photograph of the patient in the examination session: a: Lateral view showing discharge. b: A periapical radiograph showing tooth #16 with radiolucency and complete root canal filling.

She had been treated by a dermatologist and an otolaryngologist but the lesion was not cured. Then, she was referred to an endodontist. Root canal therapy of her tooth #16 was completed in two sessions, calcium hydroxide slurry was placed for a week, and the tooth was restored with amalgam. After a month, the patient returned with persistent sinus tract discharge. In clinical examination, the cutaneous lesion expressed a purulent discharge (Figure 1a).

The tooth had normal periodontal probing and mobility. The alveolar mucosa adjacent to the tooth was not tender to palpation. The patient had a non-contributory medical history.

Radiographic examination revealed periradicular radiolucencies associated with teeth #16 (Figure 1b).

The quality of the root canal filling was good radiographically. Periradicular surgery was, therefore, arranged. The patient was informed of the treatment plan and her written consent was obtained before treatment.

Chlorhexidine gluconate (0.2%) was used as a mouth rinse to reduce the number of surface microorganisms in the surgical field. After local anesthesia (2% lidocaine and 1:80000 epinephrine; Darupakhsh, Tehran, Iran) triangular full-thickness mucoperiosteal flap was reflected from teeth #14 to #17. Periapical pathological lesion was noted at all the three apices of this tooth. The granulation tissue was excised and sent for histologic evaluation. Osteotomy and apicoectomy of mesiobuccal, distobuccal, and palatal roots of tooth #16 were performed and retropreparations were made using an ultrasonic tip (E3ID; NSK, Japan). The root-end cavity was filled with mineral trioxide aggregate (ProRoot MTA; Dentsply, Tulsadental, OK, USA). All procedures were performed at high magnification (OPMI Pico Dental Microscope; Zeiss, Oberkochen, 

Germany). 

**Figure 2:**
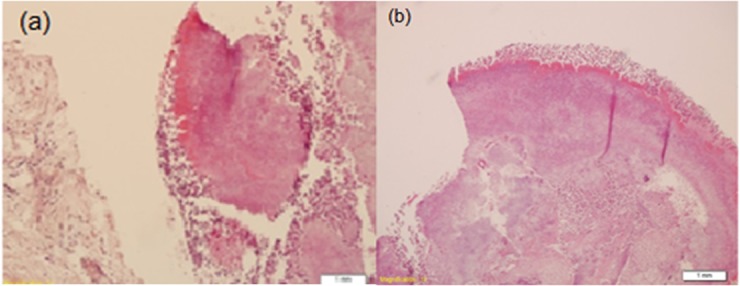
Micrograph of the lesion. a: The section shows colony of actinomycotic organisms surrounded by polymorphonuclear leukocytes with a part of connective tissue (×100). b: On higher magnification (×200), the section shows the rosette pattern of bacterial filaments. The bacterial colony has a central basophilic core and a peripheral eosinophilic portion which is surrounded by neutrophils.

The flap was sutured with 3-0 silk sutures (Ethicon; PA, USA). The patient received postoperative instructions. Postoperative analgesic (Ibuprofen 400 mg four times a day) and mouth rinse (Chlorhexidine 0.2%, 15 ml twice a day) were prescribed. The sutures were removed after four days. The patient reported a slight pain and no sign of discharge were observed. Histopathologic findings confirmed actinomycosis infection (Figure 2a, b).

**Figure 3:**
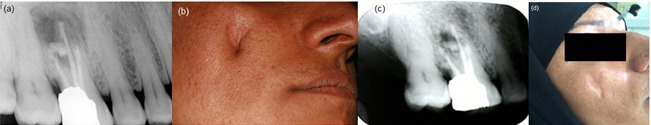
a: A radiograph after 6 months. b: A photograph in the 6-month recall. c: A radiograph after 36 months. d: A photograph after plastic surgery in the 36- month follow-up.

In the 6-month recall session, the patient had no discomfort, so she was referred to a plastic surgeon (Figure 3a, b). In the 36-month follow up, the patient was asymptomatic and revealed no sign of discharge. Radiographic examination also showed periapical healing (Figure 3c). In addition to these findings, cutaneous deficiency was corrected through plastic surgery (Figure 3d).

## Discussion

Microorganisms play an important role in the etiology and development of periradicular inflammatory responses.(1) Although nonsurgical endodontic treatment has been successful in the majority of cases, some of these inflammatory lesions do not heal by these treat ments.(9) The most common factor associated with lack of healing is the presence of microorganisms.(10)This failure is commonly related to the presence of residual bacteria (persistent infection) or reinfection of a previously disinfected root canal environment (secondary infection).(11) Actinomyces species have been reported in persistent and secondary intraradicular infections.(10)

The actinomycotic organisms are able to establish extra-radicular, so they can perpetuate even after proper root canal treatment.(8) Because they are inaccessible to endodontic disinfection procedures, these microorganisms may be a factor in the failure of root canal therapy.

If a sinus tract does not close after appropriate removal of the primary etiology, actinomycosis is the most common cause of the lesion.(12) A case report showed that actinomycosis infection may remain even after extraction of infected tooth.(13)

Post-treatment apical pathologies are managed by nonsurgical retreatment, apical surgery, or extraction and implant placement.(10) Periradicular surgery is often the last resort to maintain a root filled tooth with a persistent periapical lesion.(14)

Because of sufficient root canal treatment, it seemed that nonsurgical root canal treatment would not yield a better result and consequently, surgical treatment was indicated in this case.(15)

Periapical actinomycosis have been reported as a cause of endodontic failure;(8) however, they should not be considered a common cause of endodontic failure because of their low-prevalence infections.(16)

The incidence of this infection was reported to be 1.8% by Hirshberg* et al.*(16) and 4.4% by Nair and Schroeder.(17) Although Xia *et al.*(18) used PCR (polymerase chain reaction) and demonstrated actinomyces species to be often present in endodontic failure and their frequency to be higher than previously believed, most publications on this pathologic entity are in the form of case reports.(16, 19-20)

An odontogenic cutaneous sinus tract is a pathologic channel that originates in the oral cavity but exits at the cutaneous surface of the face or neck.(21)

Nearly 80% of the reported cases of odontogenic cutaneous sinus tracts have been associated with mandibular teeth(21-25) and cutaneous sinus tracts on the upper part of the cheek are most likely associated with the maxillary posterior teeth.(21, 23, 25)

Periapical actinomycosis is a main cause of cutaneous sinus tracts.(26) The differential diagnosis include furuncles, bacterial infections, carcinomas, osteomyelitis, pyogenic granulomas and congenital fistulas,(25) squamous cell carcinoma, thyroglossal duct cyst, branchial cleft cyst, and epidermal cyst.(27)

Due to the rarity of odontogenic cutaneous sinus tracts and the absence of dental symptoms in these patients, misdiagnosis is common.(21) Hence, the patients have multiple appointments with medical practitioners prior to a correct diagnosis.

To make the correct diagnosis, clinical and medical history, pulp and periradicular diagnostic testing, radiographic findings, tracing the sinus tract and sinus tract angiography are imperative.(25, 28)

Clinical and radiographic manifestations of actinomycosis infections are not usually distinguishable from apical periodontitis, and multiple sinus tracts are not a prerequisite for diagnosis of apical actinomycosis.(16, 19) Definitive diagnosis of this pathologic lesion is, therefore, established by histologic and microbial assessment of the surgical specimen.(20)

Strains of actinomyces are sensitive to sodium hypochlorite and calcium hydroxides, thus, prescription of antibiotics are not indicated.(29)

Although periapical surgery can resolve cutaneous sinus tract in 1 to 2 weeks,(30) fixation of the tract with the underlying tissues can cause cutaneous retraction or dimpling.(22) Therefore, the residual umbilication of the skin can be revised by cosmetic surgery.(31)

## Conclusion

This case highlights the importance of dental examination in diagnosis of a cutaneous sinus tract and consequently confirms that a decent cooperation between physicians and dentists is indispensible in better diagnosis and treatment of such challenging cases. 
